# Factors Associated with Exclusive Breastfeeding in a Maternity Hospital Reference in Humanized Birth

**DOI:** 10.1055/s-0040-1718450

**Published:** 2021-01-19

**Authors:** Gabriela Pinheiro Brandt, Alan Messala A. Britto, Camila Carla De Paula Leite, Luciana Garangau Marin

**Affiliations:** 1 Maternidade Bairro Novo, Curitiba, PR, Brazil; 2 Programa de Oncovirologia, Instituto Nacional de Câncer, Rio de Janeiro, RJ, Brazil; 3 Department of Genetics, Universidade Federal do Rio de Janeiro, Rio de Janeiro, RJ, Brazil

**Keywords:** breastfeeding, weaning, humanization, natural childbirth, cesarean section, aleitamento materno, desmame, humanização, parto normal, cesárea

## Abstract

**Objective** To analyze the factors associated with the prevalence of exclusive breastfeeding (EBF) for up to six months in mother/infant binomials cared for at a usual-risk maternity hospital.

**Methods** The present is a descriptive, longitudinal, prospective, quantitative study. Socioeconomic, obstetric and perinatal variables from 101 mother/infant binomials in a Public Maternity Hospital in the city of Curitiba, state of Paraná, Brazil, were investigated during hospitalization after delivery and 6 months after birth. For the statistical analysis, the Chi-squared test was used. The variables that showed values of *p* < 0.25 for the Chi-squared test were also submitted to an odds ratio (OR) analysis.

**Results** The prevalence (42.6%) of EBF was observed. Most women (93.1%) had had more than 6 prenatal consultations, and the variables *maternity leave* and *support to breastfeeding* were associated with EBF. Support to breastfeeding by professionals and family members increased 4-fold the chance of maintenance of EBF (OR = 0.232; 95% confidence intercal [95%CI]: 0.079 to 0.679; *p* = 0.008). Cracked nipples were the biggest obstacle to breastfeeding, and low milk production was the main responsible factor for weaning.

**Conclusion** The encouragement of breastfeeding and the mother's stay for a longer period with the child contributed to the maintenance of EBF until the sixth month of life of the infant.

## Resumo

**Objetivo** Analisar os fatores associados à prevalência do aleitamento materno exclusivo (AME) até seis meses em binômios mãe/recém-nascido atendidos em uma maternidade de risco habitual.

**Métodos** Trata-se de um estudo descritivo, longitudinal, prospectivo e quantitativo. Foram investigadas variáveis socioeconômicas, obstétricas e perinatais de 101 binômios mãe/recém-nascido de uma maternidade pública em Curitiba-PR no internamento após o parto e 6 meses após o nascimento. Para a análise estatística, utilizou-se o teste do qui-quadrado. As variáveis cujo teste do qui-quadrado tiveram valores de *p* < 0,25 foram testadas para análises de razão de probabilidades (RP).

**Resultados** Observou-se a prevalência (42,6%) do AME. A maioria das mulheres (93,1%) havia realizado mais de 6 consultas de pré-natal, e as variáveis *licença maternidade* e *apoio para amamentar* estiveram associadas ao AME. O apoio para amamentar por parte do profissional e do familiar aumentou em 4 vezes a chance da permanência em AME (RP = 0,232; intervalo de confiança de 95% [IC95%]: 0,079 a 0.679; *p* = 0,008). A fissura foi o maior obstáculo para a amamentação, e a baixa produção de leite, o principal responsável pelo desmame.

**Conclusão** O incentivo ao aleitamento e a permanência da mãe por mais tempo com a criança contribuíram para a manutenção do AME até o sexto mês de vida do bebê.

## Introduction

The World Health Organization (WHO) recommends exclusive breastfeeding (EBF) on demand, in the first six months of life, and, later, breastfeeding must be supplemented with other foods up to 2 years of age or older. 1 It is said that an infant is in EBF when he/she feeds only on breast milk, without consuming other foods or liquids. 2 This is the most complete food, and it meets the nutritional needs in the first six months of life. 3 The benefits of breastfeeding go beyond nutritional gains, as breast milk has immunological properties, favors cognitive development, and protects infants from diseases such as dehydration, diarrhea and pneumonia, which are important causes of infant mortality. 4 For the puerperal woman, it promotes the affective bond with her baby, prevents bleeding, and reduces the risk of developing cancer. 3

Increasing EBF rates are a goal to be achieved worldwide, and the WHO and the United Nations Children's Fund (UNICEF) promote and encourage the continuity of EBF. 2 3 4 5 6 In 2011, the global EBF rate in infants from 0 to 6 months was of 35%, and it increased to 40% in 2019. 2 7 In Brazil, although the EBF index is gradually increasing, its maintenance is observed for shorter periods than the recommended six months. 8

Research 3 4 6 shows that the duration and continuity of EBF are linked to socioeconomic variables such as age and maternal schooling, family income and occupation, and to obstetric and perinatal variables, such as assiduous participation in prenatal care, delivery and type of assistance received during childbirth, as well as the support provided by the professionals and family members to breastfeeding. Given the aforementioned information, the present study intended to analyze the factors associated with the prevalence of EBF for up to six months in mother/infant binomials cared for at a maternity of usual risk that is reference in good practices in care for childbirth, with Baby–Friendly Hospital Initiative (BFHI) reputation.

## Methods

The present is a descriptive, longitudinal, prospective study with a quantitative approach. It was performed in a habitual-risk public maternity hospital in the city of Curitiba, state of Paraná (PR), Brazil, a reference in humanization, with the BFHI reputation. The inclusion criteria were: women aged ≥ 18 years who gave birth to live newborns at term (≥ 37 weeks), by normal delivery or cesarean section, at the maternity hospital. Women who had premature births, stillbirths, whose newborn or themselves were transferred to high-complexity care, and who did not answer the second questionnaire were excluded.

**Fig. 1:**
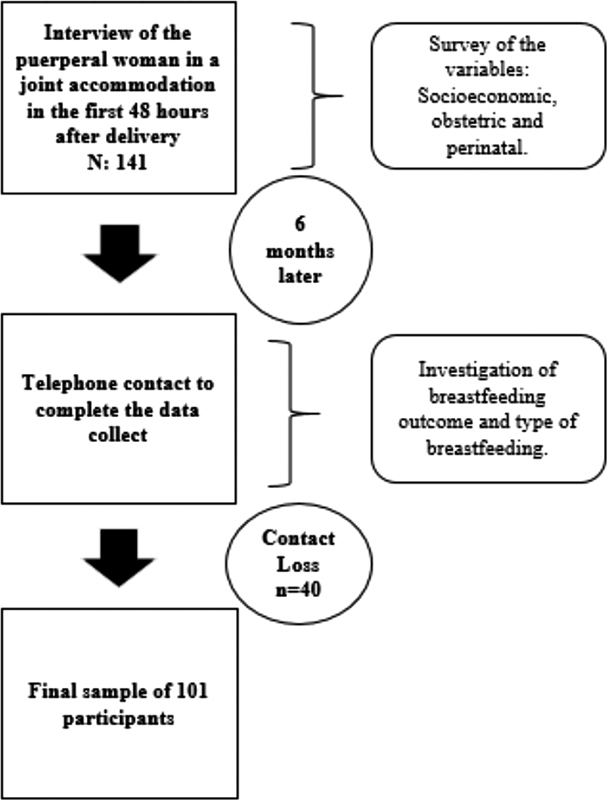
Flowchart of the data collection and selection of mother/infant binomials.

Data were collected in two moments: 1) by interview in the maternity hospital, within the first 48 hours of life, in the months of January and February 2019; and 2) through a phone call with the mother, at 6 months of life of the infant, in August 2019. The collection was prospective and used 2 structured questionnaires, composed of 12 and 10 questions respectively, prepared by the researchers and previously tested. In the first questionnaire, socioeconomic, obstetric and perinatal variables were collected, while in the second, we collected information about the duration of the EBF and the type of breastfeeding the infant was on at six months. Fig. 1 shows the flowchart of the data collection and the selection of mother/infant binomials based on the inclusion and exclusion criteria.

In the first contact, the women who agreed to participate signed the free and informed consent form (FICF), with a total of 141 participants. Telephone contact was obtained with only 101 participants in the second collection, even after 3 attempts to call at different dates and times. We analyzed the outcome and the type of breastfeeding according to the classification by the WHO: EBF, when the infant is fed only breast milk, without the addition of other foods or liquids; breastfeeding (BF), when, in addition to breast milk, the infant is fed other liquid and/or solid foods; and mixed breastfeeding (MBF), when the infant is fed breast milk and baby formula. 9

We collected socioeconomic variables (age and maternal schooling, family income, occupation and maternity leave), obstetric variables (type of delivery, parity, and number of prenatal consultations), and variables related to good perinatal practices (skin-to-skin contact [when the infant stays with the mother immediately after the birth for at least 1 hour], breastfeeding in the first hour of life, and support to breastfeeding from a professional or family member) to look for an association with EBF. Regarding the variable *maternal schooling* , illiterate women and those with incomplete elementary education were included in the ‘less than 8 years of schooling’ group, while those with complete elementary education up to complete higher education were included in the ‘more than 8 years of schooling’ group. The factors that made breastfeeding difficult and the factors that motivated weaning were also analyzed.

The information was tabulated in Excel 2016 (Microsoft Corp., Redmond, WA, US) spreadsheets, and the statistical analysis was performed using the Statistical Package for the Social Sciences (SPSS, IBM Corp., Armonk, NY, US), version 21.0. The absolute and relative frequencies were calculated, in addition to the search for an association of the variables with EBF through the chi-squared test of independence, in which values of *p* < 0.05 were considered significant. The variables that had values of *p* < 0.25 in the Chi-squared test were tested for an analysis of the odds ratio (OR) using the MedCalc web site ( https://www.medcalc.org/calc/odds_ratio.php ). The present research was submitted to analysis and approved by the Ethics in Research Committee of the municipality of Curitiba (under opinion No. 3,060,900 on December 6, 2018).

## Results

Overall, 101 mother/newborn binomials were interviewed, most of which were still breastfeeding (74.3%) ( Table 1 ). As for the type of breastfeeding at six months of life, 42.6% remained on EBF, and almost a third of the sample continued to breastfeed, but not exclusively (BF = 18.8%; MBF = 12.9%), and only 25.7% of the infants weaned early ( Table 1 ).

**Table 1:** Breastfeeding outcome ( *n* = 101)

Variable		n	%
Breastfeeding	Yes	75	74.3
	No	26	25.7
Breastfeeding type	Exclusive breasfeeding	43	42.6
	Breastfeeding	19	18.8
	Mixed breastfeeding	13	12.9
	Weaning	26	25.7

Regarding the characteristics of the population, the most prevalent maternal age group was 20 to 34 years (80.2%), and just over 80% of the mothers had more than 8 years of schooling ( Table 2 ). It is noteworthy that there were no illiterate women, and that 71.3% of them had at least graduated from High School. The most frequent family income was more than 2 minimum wages (74.3%), and half of the women reported contributing to the houehold income, since they worked (49.5%; Table 2 ). As for the employment relationship, 36.6% were employed with a formal contract, and 12.9% declared themselves self-employed. Regarding maternity leave, 38.6% enjoyed a period of 4 to 6 months of maternity leave. Most women were primiparous (47.5%), had a normal birth (73.3%), and had regular prenatal care with more than 6 consultations (93.1%). Regarding good practices, skin-to-skin contact stood out as the experience most lived by women (78.2%), which results in a good rate of breastfeeding in the first hour of life (65.3%). Also noteworthy is the high prevalence of ‘support to breastfeeding’ (74.3%), showing the engagement of the team and family members in breastfeeding. This support was defined as a set of practices and information that the puerperal woman received from the multiprofessional team during hospitalization, and, later, the support she received at home from the family to continue breastfeeding.

**Table 2:** Association of exclusive breastfeeding and socioeconomic, obstetric and perinatal variables ( *n* = 101)

Variable		Exclusive breastfeeding		*p-value*	Total
		Yes	No		
		n (%)	n (%)		n (%)
		43	58		101
Age	18–19 years	4 (9.3)	5 (8.6)	0.96	9 (8.9)
	20–34 years	34 (79.1)	47 (81.0)		81 (80.2)
	> 35 years	5(11.6)	6 (10.4)		11 (10.9)
Schooling	< 8 years	11(25.6)	8 (13.8)	0.71	19 (18.8)
	> 8 years	32 (74.4)	50 (86.2)		82 (81.2)
Family income	≤ 2 minimum wages	9 (20.9)	17 (29.3)	0.56	26 (25.7)
	> 2 minimum wages	34 (79.1)	41 (70.7)		75 (74.3)
Currently employed	Yes	19 (44.2)	31 (53.4)	0.26	50 (49.5)
	No	24 (55.8)	27 (46.6)		51 (50.5)
Maternity leave	Yes	13 (30.2)	26 (44.8)	0.02*	39 (38.6)
	No	30 (69.8)	32 (55.2)		62 (61.4)
Birth type	Normal	30 (69.8)	44 (75.9)	0.31	74 (73.3)
	Cesarean section	13 (30.2)	14 (24.1)		27 (26.7)
Parity	First pregnancy	19 (44.2)	29 (50.0)		48 (47.5)
	2–3 pregnancies	21 (48.8)	24 (41.4)	0.75	45 (44.6)
	≥ 4 pregnancies	3 (7.0)	5 (8.6)		8 (7.9)
Prenatal consultation	< 6	3(7.0)	4 (6.9)	0.98	7 (6.9)
	≥ 6	40 (93.0)	54 (93.1)		94 (93.1)
Skin-to-skin contact	Yes	30 (69.8)	49 (84.5)	0.07	79 (78.2)
	No	13 (30.2)	9 (15.5)		22 (21.8)
Breastfeeding in the first hour	Yes	26 (60.5)	40 (69.0)	0.37	66 (65.3)
	No	17 (39.5)	18 (31.0)		35 (34.7)
Support to breastfeeding	Yes	38 (88.4)	37 (63.8)	0.005*	75 (74.3)
	No	5 (11.6)	21 (36.2)		26 (25.7)

**Source:** Data of the survey, 2019.

Note: * *p* < 0.05.

The comparison between the mother/infant binomials who maintained EBF with those that did not, and the association with the socioeconomic, obstetric and perinatal variables were performed using the Chi-squared test ( Table 2 ). Regarding the socioeconomic variables, only maternity leave was statistically different among the groups. Contrary to expectations, the women who did not take maternity leave maintained EBF for longer periods when compared with those who took leave ( *p* = 0.02). The obstetric variables *type of delivery* , *prenatal consultations* , and *parity* did not present a statistically significant difference among the women who maintained EBF or not at six months ( Table 2 ). As for the variables related to good perinatal practices, EBF was more prevalent among the women who received support to breastfeed than among the women who did not maintain EBF ( *p* = 0.005), and the variable *skin-to-skin contact* , despite not having presented a statistically significant difference, tended to be lower among the mother/infant binomials who maintained EBF.

Then, we evaluated whether the variables *maternity leave* , *support to breastfeeding* (which were associated with breastfeeding) and *skin-to-skin contact* (which tended to be associated with breastfeeding) were risk or protective factors for EBF through the calculation of the OR. This analysis showed that taking maternity leave tended to increase the probability of maintenance of the EBF (OR = 0.533; 95% confidence interval [95%CI]: 0.232 to 1.225; *p* = 0.138), and skin-to-skin contact tended to decrease this probability (OR = 2.359; 95%CI: 0.90 to 6.1845; *p* = 0.081). In contrast, professional and family support to breastfeeding increased the chance of breastfeeding 4-fold (OR = 0.232; 95%CI: 0.079 to 0.679; *p* = 0.008).

Finally, the factors that made breastfeeding difficult and that influence weaning were investigated ( Table 3 ). Approximately half of the interviewees (46.5%) reported some difficulty in breastfeeding, the most predominant being ‘nipple fissure’ (22.8%), followed by the complaint of low ‘milk production’ (17.8%). Weaning affected 25.7% of the population, and low milk production appears as the main driver (42.3%), followed by weaning by maternal option (30.8%) and return to work (26.9%).

**Table 3:** Main difficulties with breastfeeding and reasons for weaning ( *n* = 101)

Variable		n	%
Difficulty breastfeeding	No	54	53.5
N = 101	Fissure	23	22.8
	Mastitis	5	5.0
	Engorgement	1	1.0
	Low milk production	18	17.8
Reason for weaning N = 26	Return to work	7	26.9
	Low milk production	11	42.3
	By option	8	30.8

**Source:** Research data, 2019.

## Discussion

In view of the benefits for the mother/infant binomial and the WHO recommendations regarding the maintenance of EBF in the first six months of life of the infant, the aim of the present study was to describe the socioeconomic, obstetric and perinatal aspects related to childbirth care that influenced EBF in an usual-risk maternity, a reference in good practices in childbirth and birth care. There was a high rate of breastfeeding (73.4%) among the population studied, in addition to EBF rates (42.6%) above the data estimated for Brazil (38.6%) and the world (40%), according to data from the UNICEF. 7 In Brazil, EBF rates have been gradually increasing, and although they are almost twice as high as those in middle- and upper-income countries (23.9%), they are still far behind the rates of countries like Rwanda (86.9%), Burundi (82.3%) and Sri Lanka (82%), which have the highest EBF rates in the world. 7 In the state of Pernambuco, Brazil, a study revealed that the median period of EBF was of only 60.84 days, which indicates that the good practices of the institution studied here and the BFHI seem to have positively influenced the maintenance of EBF. 8

Age is one of the factors that can affect EBF. Some authors believe that women over the age of 30 breastfeed longer than younger women, and that adolescence can be a weaning factor. 3 4 In the present study, most women were aged between 20 and 34 years, but age was not associated with EBF. Likewise, the EBF was not related to schooling. There are reports that mothers with more than eight years of schooling breastfeed more, as they have more access to information. 3 In addition, the low level of schooling can also delay the beginning of prenatal care, which directly results in successful breastfeeding. 3 In contrast, the higher level of schooling of the women can increase the rate of formal employment and result in an early return to work, which influenced the early weaning of 7 of the 26 patients who stopped breastfeeding before 6 months. 8

In the present study, women who did not take maternity leave breastfed more. There are studies that state that the mother's presence at home is positive for the continuity of EBF, while others claim the opposite. 3 10 In the present study, all unemployed women belonged to the group who did not receive maternity leave, so we believe that staying at home for this population is a factor that protects breastfeeding.

Regular prenatal care with more than six consultations and mainly with quality of care and guidance is a greatly for the success of breastfeeding. 2 8 A high adherence to prenatal care was observed in the studied group (93% had had ≥ 6 consultations). A longitudinal study 2 with 531 infants in 2012 found that the absence of prenatal care increased the chance of reducing breastfeeding time by 173%.

The studied population had a high rate of normal delivery (73.3%), the most recommended route for birth by the WHO. Cesarean section, in turn, is considered a hindrance to breastfeeding in the first hour of life, a variable that has already been related to the longer duration of breastfeeding. 3 8 Although two thirds of the mothers studied had breastfed in the first hour of life, there was no association between this variable and EBF.

A Cochrane review 13 sought randomized trials on skin-to-skin contact and breastfeeding, and concluded that mothers who had skin-to-skin contact breastfed exclusively for longer periods. 5 6 7 8 9 10 11 12 13 Although skin-to-skin contact was more frequent among women who weaned in this particular sample, the practice is encouraged by the WHO, and it corresponds to step four of the *Ten Steps to Successful Breastfeeding* in the BFHI. 12

Women who received ‘support to breastfeeding’' were 4 times more likely to maintain EBF ( *p* = 0.008). The support network for the puerperal woman must start in the prenatal period, and remain during the care received at the hospital and after discharge, since, due to the difficulties that arise during the breastfeeding period, the puerperal woman can seek support and continue to breastfeed. 6 The support of the partner in this network reinforces the importance of family members involvement in the whole process of gestating, giving birth and maternal. The importance of the team in maintaining EBF during hospitalization at the maternity hospital is highlighted, as the mother/infant binomials discharged on EBF are 2.5 times more likely to maintain the EBF. 3 4 5 6 7 8 9 10 11 12 13 14 The hospital where the present study took place offers support through the promotion, protection and encouragement of breastfeeding during the entire hospitalization. This is done through guidance and assistance in breastfeeding, added to the good practices of care during childbirth. The maternity in question has an Interdisciplinary Committee on Breastfeeding, which is composed of an engaged multidisciplinary team (doctors, nurses, nutritionist, social worker and speech therapist) and promotes courses, workshops, lectures, research and discussions in this field, with the objective of supporting breastfeeding and increasing breastfeeding rates. The assistance team works with individualized care and daily physical examination of the breasts to identify nipple fissures, engorgement and solve the doubts of the women during the entire hospitalization. The maternity hospital also has an exclusive breastfeeding support room, a pleasant and reserved place, which is ideal for individualized and differentiated care. All women should be instructed on the importance of breastfeeding, on the correct position to breastfeed, on milking the breasts when necessary, and on the prevention of fissures and other complications, and as to when to seek help and professional support.

Difficulties in breastfeeding usually occur in cascade. The position of the mother/infant binomial affects the grip and suction, which can result in nipple fissure that generates pain. 5 Due to pain, the puerperal woman tends to offer the breast less often to the infant, which increases the likelihood of low milk production or results in breast engorgement. 15 Nipple fissure, the most frequent complaint in this population, is seen in the literature as an important factor for weaning. 15 Although many women have reported insufficient milk production, it is known that, biologically, the production is sufficient for their children. This statement denotes the insecurity that usually disappears over time, if the mother receives adequate guidance and support. 6

The bias of postpartum memory failure and the fact that the second part of the data collection was performed by telephone, which may allow for some misunderstanding in the questions used, are the main limitations of the present study. Thus, it is necessary to conduct new studies with the local population, and to compare different institutions to promote current results that strengthen breastfeeding assistance.

## Conclusion

The factors that were associated with the duration of EBF in the present study were staying at home with the child longer, and the support of the professional or family members to breastfeeding, which reduced the chance of interrupting EBF four-fold. Although the other variables discussed here are not significant, it is known that good practices reflect on all of assistance provided and throughout the life of the mother/infant binomial. Finally, data on the factors associated with early weaning provide a basis to support interventions and discussions capable of improving the quality of care for the maternal and infant population.

## References

[1] World Health Organization Guideline: protecting, promoting and supporting breastfeeding in facilities providing maternity and newborn services [Internet] Geneva WHO 2017[cited 2019 Oct 20]. Available from:http://www.who.int/nutrition/publications/guidelines/breastfeeding-facilities-maternity-newborn/en/29565522

[2] Ferreira H LOC Oliveira M F Bernardo E BR Almeida P C Aquino P S Pinheiro A KB Factors associated with adherence to exclusive breastfeeding Cien Saude Colet 2018 23 03 683 690 10.1590/1413-81232018233.06262016 29538549

[3] Margotti E Margotti W [Factors related to exclusive breastfeeding in babies born in a child-friendly hospital in a northern Brazilian capital] Saúde Debate 2017 41 114 860 871 10.1590/0103-1104201711415

[4] Cavalcanti S H Caminha MdeF Figueiroa J N Serva V MSBD Cruz R SBLC de Lira P IC Batista Filho M Factors associated with breastfeeding practice for at least six months in the state of Pernambuco, Brazil Rev Bras Epidemiol 2015 18 01 208 219 10.1590/1980-5497201500010016 25651022

[5] Carreiro J A Francisco A A Abrão A CFV Marcacine K O Abuchaim E SV Coca K P Breastfeeding difficulties: analysis of a service specialized in breastfeeding Acta Paul Enferm 2018 31 04 430 438 10.1590/1982-0194201800060

[6] Amaral L JX Sales SdosS Carvalho D PSRP Cruz G KP Azevedo I C Ferreira Júnior M A [Factors that influence the interruption of exclusive breastfeeding in nursing mothers] Rev Gaúcha Enferm 2015 36(Spec No):127 134 10.1590/1983-1447.2015.esp.56676 27057711

[7] United Nations Organization [UNICEF: only 40% of children in the world receive exclusive breastfeeding early in life] [Internet] New York United Nations 2019[cited 2019 Oct 15]. Available from:https://nacoesunidas.org/unicef-apenas-40-das-criancas-no-mundo-recebem-amamentacao-exclusiva-no-inicio-da-vida/

[8] Santos E MD Silva L SD Rodrigues B FS de Amorim T MAX da Silva C S Borba J MC Tavares F CLP [Breastfeeding assessment in children up to 2 years of age assisted in primary health care of Recife in the state of Pernambuco, Brazil] Cien Saude Colet 2019 24 03 1211 1222 10.1590/1413-81232018243.126120171 30892540

[9] Ministério da Saúde Secretaria de Atenção à Saúde Departamento de Atenção Básica [Child health: breastfeeding and complementary feeding] Brasília (DF) Ministério da Saúde 2015

[10] Campos A MS Chaoul CdeO Carmona E V Higa R do Vale I N Exclusive breastfeeding practices reported by mothers and the introduction of additional liquids Rev Lat Am Enfermagem 2015 23 02 283 290 10.1590/0104-1169.0141.2553 26039299 PMC4459002

[11] Silva C M Pereira S CL Passos I R Santos L C [Factors associated with skin-to-skin contact between mother / child and breastfeeding in the delivery room] Rev Nutr 2016 29 04 457 471 10.1590/1678-98652016000400002

[12] D'Artibale E F Bercini L O The practice of the fourth step of the baby-friendly hospital initiative Esc Anna Nery 2014 18 02 356 364 10.5935/1414-8145.20140052

[13] Moore E R Bergman N Anderson G C Medley N Early skin-to-skin contact for mothers and their healthy newborn infants Cochrane Database Syst Rev 2016 11 11 CD003519 10.1002/14651858.CD003519.pub4 27885658 PMC6464366

[14] Cruz N ACV Reducino L M Probst L F Guerra L M Ambrosano G MB Cortellazzi K L [Association between the type of breastfeeding at discharge of the newborn and at six months of life] Cad Saude Colet 2018 26 02 117 124 10.1590/1414-462x201800020349

[15] Moraes B A Gonçalves A C Strada J KR Gouveia H G Factors associated with the interruption of exclusive breastfeeding in infants up to 30 days old Rev Gaúcha Enferm 2017 37(spe):e2016-0044 10.1590/1983-1447.2016.esp.2016-0044 28746498

